# The Role of Alveolar Edema in COVID-19

**DOI:** 10.3390/cells10081897

**Published:** 2021-07-26

**Authors:** Shu Yuan, Si-Cong Jiang, Zhong-Wei Zhang, Yu-Fan Fu, Jing Hu, Zi-Lin Li

**Affiliations:** 1 College of Resources, Sichuan Agricultural University, Chengdu 611130, China; zzwzhang@126.com (Z.-W.Z.); stefanlife@126.com (Y.-F.F.); 2 Chengdu Kang Hong Pharmaceutical Group Comp. Ltd., Chengdu 610036, China; jiangsc@cnkh.com; 3 School of Medicine, Northwest University, Xi’an 710069, China; hujinglzbx@163.com; 4 Department of Cardiovascular Surgery, Xijing Hospital, Medical University of the Air Force, Xi’an 710032, China; lizhuoyuanbb@163.com

**Keywords:** SARS-CoV-2, endothelial injuries, ventilation-perfusion mismatch, intravascular coagulation, alveolar edema, oxygen therapy

## Abstract

The coronavirus disease 2019 (COVID-19) has spread over the world for more than one year. COVID-19 often develops life-threatening hypoxemia. Endothelial injury caused by the viral infection leads to intravascular coagulation and ventilation-perfusion mismatch. However, besides above pathogenic mechanisms, the role of alveolar edema in the disease progression has not been discussed comprehensively. Since the exudation of pulmonary edema fluid was extremely serious in COVID-19 patients, we bring out a hypothesis that severity of alveolar edema may determine the size of poorly-ventilated area and the blood oxygen content. Treatments to pulmonary edema (conservative fluid management, exogenous surfactant replacements and ethanol–oxygen vapor therapy hypothetically) may be greatly helpful for reducing the occurrences of severe cases. Given that late mechanical ventilation may cause mucus (edema fluid) to be blown deep into the small airways, oxygen therapy should be given at the early stages. The optimal time and blood oxygen saturation (SpO_2_) threshold for oxygen therapy are also discussed.

## 1. Introduction

Since the outbreak of novel SARS-like coronavirus (SARS-CoV-2), over 181,521,067 confirmed cases and 3,937,437 confirmed deaths have been reported globally by the World Health Organization (WHO) as of 1 July 2021. Acute respiratory distress syndrome (ARDS) and the serious complications (mainly multiple organ failure) are the most frequent causes of death [1,2,3,4]. However, the main cause of death was not direct ARDS, but the multiple organ failure, such as RNAemia, acute cardiac injury, acute renal injury and septic shock [1,2,3,4]. ARDS is a type of respiratory failure whose definition is based on a ratio of arterial oxygen tension to fractional inspired oxygen (*P*_a_O_2_:*F*_i_O_2_) of less than 300 mm Hg despite a positive end-expiratory pressure of more than 5 cm H_2_O [5,6], which means that hypoxemia is a necessary condition for the patient to have an ARDS. A longer-time hypoxemia may result in cardiac ischemia, renal ischemia or RNAemia/septic shock, indicating a multiple organ ischemia [1,2,3,4]. On the other hand, some features of COVID-19 pneumonia distinguish it from typical ARDS. The patients at early stages often display little breathlessness, despite profound hypoxemia, a symptom referred to as “happy hypoxemia”. A report showed dyspnea in only 18.7% of 1099 hospitalized COVID-19 patients, despite their low ratios of partial pressure of arterial oxygen to percentage of inspired oxygen; in contrast, as high as 86% of the patients showed abnormal computerized tomography scans [7]. Although the profound hypoxemia was associated with a large intrapulmonary shunt (also different from typical ARDS), alveolar cells are well preserved in COVID-19 relative to typical ARDS [8].

In addition to the need for oxygen supply in a high proportion (41%) of COVID-19 patients [7], 56% of the patients admitted to intensive care unit (ICU) were given non-invasive ventilation, 76% of whom required further orotracheal intubation and invasive mechanical ventilation [9]. Nevertheless, in some underdeveloped countries, there is extraordinary shortage of ventilators and extracorporeal membrane oxygenation (ECMO), which may lead to higher mortality rates. More efficient therapies to improve patients’ breathing or treatments that could reduce the probability of the occurrence of severe cases need to be developed immediately.

## 2. Endothelial Injuries in COVID-19

The multiple roles of alveolar endothelial and epithelial barriers have been well documented in many lung diseases, such as acute lung injury (ALI), idiopathic pulmonary fibrosis (IPF) and chronic obstructive pulmonary disease (COPD) [5]. However, the role of alveolar injuries in the disease progression of COVID-19 has not been discussed comprehensively so far [10,11].

During the mechanism of dysfunction of the alveolar epitheliums, important roles are related to alveolar epithelial cells type I/II (AEI/AEII). AEII coordinates the host defense mechanisms, not only generating the restrictive alveolar epithelial barrier, but also secreting pulmonary surfactant, which reduces surface tension at the pulmonary air–liquid interface, thereby preventing atelectasis and alveolar edema [10,11,12,13,14]. Furthermore, the innate immune responses to infection of AEII lead both to the cell death by pyroptosis and apoptosis and to the activation alveolar macrophages [10,11,12,13,14]. In addition, the glucocorticoid receptor α acts as a cellular rheostat to ensure that a proper response is elicited by the neuro-endocrine and immune systems [6,15]. Actually, various chronic and acute diseases (including COVID-19) are associated with intensive inflammations [1,2]. Hyperplastic AEII are considered to be an essential part of the epithelialization processes and, consequently, endothelial injury healing [10,11,12,13,14].

The lung’s initial response to acute viral infections has been characterized by innate immunity-mediated damages of the alveolar endothelial and epithelial barriers and accumulation of protein-rich edema fluid within the interstitium and alveolus, and then a great decline in oxygen diffusion over the blood-air barrier [5]. Blood flow through severely-damaged units, hereby, constitutes an intrapulmonary shunt and the hypoxia [5]. Acute hypoxia inhibits Na/K-ATPase function by activating its endocytosis from the plasma membrane to intracellular compartments. The endocytosis process is suggested to be mediated by the accumulation of reactive oxygen species (ROS) in mitochondria. Then ROS promotes the protein kinase C (PKC)-zeta dependent phosphorylation of the Na/K-ATPase α subunit triggering its endocytosis in a clathrin-AP2 dependent pathway [16]. More prolonged hypoxia may cause the ubiquitination and degradation of Na/K-ATPase subsequently. As a result, hypoxia inhibits K^+^ channels but activates voltage gated Ca^2+^ channels, which raises cytosolic Ca^2+^ levels in pulmonary artery smooth muscle cells (PASMC) and causes vasoconstriction [5,17]. Thus at the tissue level, patients of acute lung injury often develop the hypoxic pulmonary vasoconstriction (HPV), which is an essential protection mechanism of the lung that directs blood perfusion from badly-ventilated to well-ventilated alveoli to optimize gas exchange [18,19]. Endothelin-1 and thromboxane A_2_ may amplify, whereas prostacyclin and nitric oxide (NO) may moderate this process [19]. In addition, prostacyclin was suggested to be coupled mainly to cyclooxygenase-1 in acute hypoxia, but to cyclooxygenase-2 in chronic hypoxia [19].

At the subcellular level, mitochondria in PASMC have been considered to be oxygen sensors and initiate HPV [20,21,22,23,24]. Warburg metabolism (a phenomenon firstly found in tumor cells, which almost exclusively use glycolysis to generate energy, even under aerobic conditions) in PASMC mitochondria is initiated by the induction of a pseudo-hypoxic state, where DNA methyltransferase-mediated regulation in redox signaling results in normoxic activation of hypoxia inducible factor-1α (HIF-1α) and pyruvate dehydrogenase kinase (PDK) accumulation [20,21,22,23,24]. On the other hand, mitochondrial division has been also proved to be coordinated with nuclear division via the cellular process named mitotic fission. Increased mitotic fission in HPV, driven by enhanced fission but repressed fusion, accelerates the cell cycle and increases apoptosis resistance [20,21,22,23,24]. Thus, Warburg metabolisms sustain energy homeostasis through inhibiting oxidative metabolism, which may reduce mitochondrial apoptosis, and lead to uncontrolled cell division. Mitochondrial dynamic and metabolic disorders combine to show the hyper-proliferative and apoptosis-resistant phenotypes in pulmonary artery smooth muscle cells [20,21,22,23,24].

The renin-angiotensin-aldosterone system (RAAS) also plays a key role in endothelial injuries. RAAS is a key regulatory system of electrolyte homeostasis and pulmonary artery status and functions through angiotensin-converting enzyme (ACE)/angiotensin II (Ang II)/Ang II type 1 (AT1) receptor axis and angiotensin-converting enzyme 2 (ACE2)/angiotensin-1–7 Ang-(1–7)/MAS receptor axis. RAAS dysfunction has been proved to be related to the occurrence and development of acute pulmonary injuries and ARDS and may cause a serious prognosis and even death [25,26,27,28,29]. A large number of studies suggest that ACE2 works as the cellular receptor for SARS-CoV-2 entry [30,31,32]. Similar to other families of Coronaviridae viruses, the protein interactions between the virus and the membrane-bound ACE2 requires the cleavage of the spike glycoprotein S into two subunits. After the binding of the S1 receptor-binding domain (RBD) to ACE2, the trans-membrane protease/serine subfamily 2 (TMPRSS2) cleaves the S2 subunit and facilitates membrane fusion [30,31,32]. It has been confirmed that SARS-CoV-2 cell entry results in decline of the ACE2 protein, which converts Ang II into Ang-(1–7). Ang II induces pro-inflammatory cascades when binding to the AT1 receptor, while Ang-(1–7) generates anti-inflammatory effects through the interaction with the MAS receptor. Thus, down-regulation of ACE2 would result in pulmonary injury and vasoconstriction [25,26,27,28,29,33]. Besides these mechanisms, relative ACE2 deficiency may also lead to enhanced and protracted tissues, and vessel exposure to Ang II, which then enhances thrombosis, cell proliferation and recruitment, increases tissue permeability, cytokine production and results in inflammation (Table 1) [33,34].

## 3. Ventilation-Perfusion Mismatch and Intravascular Coagulation in COVID-19

HPV is thought to optimize gas exchange through shunting blood from poorly ventilated areas to those rich in oxygen. However, a general pulmonary vasoconstriction happens in both normoxic lung competencies and hypoxic lung competencies, which may lead to the pulmonary hypertension (PH) and a risk of right-heart failure subsequently, just as observed in patients with acute altitude sickness [19,35,36]. HPV has been observed in COVID-19 patients [37,38]. Moreover, the pulmonary hypertension and the subsequent right ventricular dysfunction also have been confirmed in severe COVID-19 patients (Table 1) [39,40]. Significant alternations in monocyte size, lymphocyte stiffness, neutrophil size and deformability, and heterogeneity of erythrocyte size and deformation in COVID-19 patients also indicated great changes in pulmonary blood stream dynamics [41].

Nevertheless, drugs to inhibit HPV, such as acetazolamide, calcium channel blockers and phosphodiesterase-5 inhibitors, should be avoided for COVID-19 patients, given that the consequences of COVID-19 may be exacerbated by loss of HPV, whether because of the destructive effects of the virus on mitochondria or the ability of endotoxin and inflammatory stimuli to eliminate HPV [38]. HPV attenuating drugs may exacerbate hypoxemia in COVID-19 pneumonia [38].

In some cases, dysregulated HPV may also cause mismatched blood flow and alveolar ventilation, which may restore the oxygen supply to the poorly-ventilated alveolar cells and result in life-threatening hypoxemia [35]. Recently, Herrmann et al. [42] modeled lung perfusion abnormalities and suggested that early COVID-19 hypoxemia may be mainly attributed to severe ventilation-perfusion mismatch. Their model predicted that calculated shunt fractions in excess of three times the injured fractions, which could be explained by (a) extensive perfusion defect, (b) perfusion defect combined with ventilation-perfusion mismatching in the non-injured fraction, or (c) hyper-perfusion of the small injured regions, with up to 3-fold increases in regional perfusion to the afflicted fraction [42]. Clinical features of COVID-19 pneumonia also confirmed the malfunction of oxygen-sensing responses including the ventilation-perfusion mismatch (Table 1) [43].

Pulmonary intravascular coagulation also plays an important role in the disease progression of COVID-19. After alveolar injuries, resident alveolar macrophages are activated, causing the release of potent proinflammatory mediators and chemokines that promote the accumulation of neutrophils and monocytes, such as vascular endothelial growth factor (VEGF), angiotensin II (Ang II), glycosaminoglycans (GAGs), von Willebrand factor (vWF) and soluble intercellular adhesion molecule (sICAM-1) [5]. Activated neutrophils further contribute to injuries through releasing toxic mediators. On the other hand, intravascular coagulation leads to platelet aggregation and micro-thrombi formation, which may aggravate the pulmonary injuries [5]. Intravascular coagulation primarily leads to increased dead space (increased wasted ventilation and less efficient carbon dioxide removal). Whereas inflammatory mediators from endothelial injury may worsen hypoxemia through exacerbate ventilation-perfusion mismatching (Table 1) [35,44]. Coagulation and thrombosis have been identified clinically as prominent symptoms of COVID-19 [45,46].

Nevertheless, neither intravascular coagulation nor ventilation-perfusion mismatch could be easily corrected. Oxygen supply is a common treatment to COVID-19 patients. Increasing inspired oxygen results in enhanced oxygenation but however does not improve the ratio of arterial oxygen tension to fractional inspired oxygen (*P*_a_O_2_:*F*_i_O_2_) [42]. Inhaled nitric oxide decreases total pulmonary vascular resistance [47,48] but however may increase blood flow to these low ventilation-perfusion regions, which causes further arterial desaturation (Table 2) [35].

Aspirin, the most commonly used anti-platelet agent, is a cyclooxygenase-1 inhibitor and considered as a mild to moderate inhibitor of platelet function [49,50]. Hereby, aspirin has been suggested for COVID-19 patients [49,50]. Aspirin administration had an association with less mechanical ventilation and reduced ICU cases, but showed no apparent association with the fatality rate (Table 2) [51]. The therapeutic effects of low molecular weight heparin have also been investigated comprehensively [52,53,54,55,56,57,58]. Heparin may reduce the risk of in-hospital mortality and decrease the occurrence of severe cases. However, critically-ill COVID-19 patients still had high incidences of venous thromboembolism and worse outcomes, despite the heparin administration at the prophylactic dosage [52,53,54,55,56,57,58]. Moreover, besides its anticoagulation effect, heparin relieves the symptoms through complex mechanisms. SARS-CoV rolls onto the cell membrane by binding to cell-surface heparan sulfate proteoglycans (HSPGs) and scans for the specific entry receptor ACE2 [59,60]. In addition, heparin may enhance the open conformation of the subsequent ACE2 binding. Thus, heparin potently blocks both viral adhesion and spike protein binding with the host cell plasma membrane [59,60]. On the other hand, an heparin-binding sequence immediately upstream of the S1/S2 cleavage site has been found on SARS-CoV-2 S protein, indicating that heparin may promote the S1/S2 cleavage, induce exposure of the optimal epitope, and therefore accelerate the virus clearance [61]. These assumptions have been proved by a serological study that adding 10 μM heparins into the sera from COVID-19 patients led to a four-fold increase in antibody titers [62].

In summary, initial anticoagulant treatments with low-molecular-weight heparin or aspirin may reduce mortality and achieve a significant improvement in *P*_a_O_2_:*F*_i_O_2_ in some patients but however could not completely prevent occurrence of severe cases [49,50,51,52,53,54,55,56,57,58].

## 4. Alveolar Edema in COVID-19

Besides above pathogenic mechanisms, alveolar edema also plays a key role in the disease progression. Diffuse alveolar damage (DAD) is the histopathological pattern commonly described in COVID-19 (Table 1) [63,64,65]. Endothelial barrier disruption induces interstitial flooding via activation of the actin-myosin contractile apparatus [5]. Then alveolar edema leads to hypoxia at the injured alveolar units [5]. Hypoxia in turn inhibits edema fluid clearance, due in part to the disassembly of the keratin intermediate filament network, a fundamental element of the cellular cytoskeleton, therefore destructing the epithelial barrier [66]. Therefore, a long-term hypoxia aggravates the disease by inducing more alveolar edema, which forms a vicious circle (Table 1).

**Figure 1:**
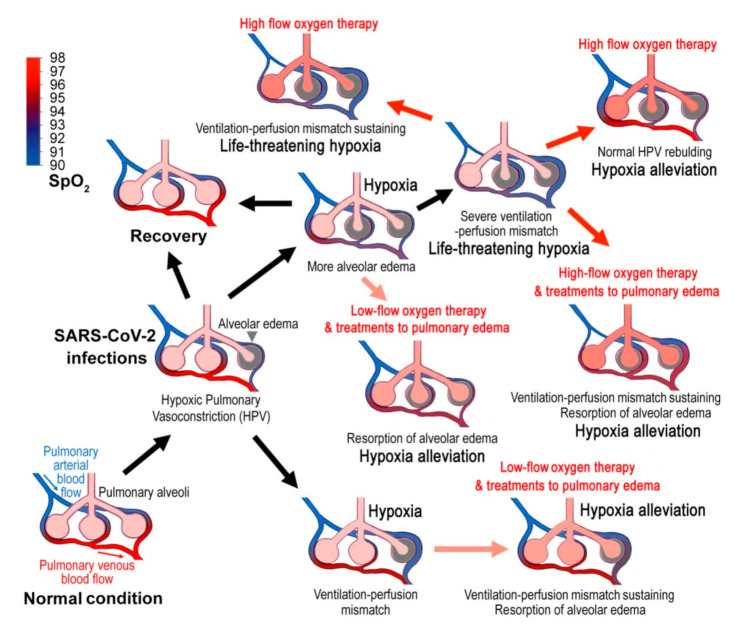
Hypothetically pathogenic mechanisms of COVID-19 and the corresponding therapies. Virus infections cause alveolar edema and the hypoxic pulmonary vasoconstriction (HPV), which is an essential protection mechanism of the lung that directs blood perfusion from badly-ventilated to well-ventilated alveoli to optimize gas exchange. However, SARS-CoV-2 infections may cause mismatched blood flow and alveolar ventilation, and may result in life-threatening hypoxemia. Treatments to pulmonary edema might promote lung edema resorption and show good therapeutic effects. When the disease develops into late stages, systemic alveolar edema and severe ventilation-perfusion mismatch occurs, and the blood oxygen will decline sharply. Then the high-flow oxygen therapy will be required. However, high-flow oxygen inhalation may not correct ventilation-perfusion mismatch and cause several adverse effects. The blue to red gradient bar shows blood oxygen saturation (SpO_2_).

Impaired alveolar surfactant production may be another molecular mechanism of alveolar edema in COVID-19 in that the limitation of alveolus superficial active substance would increase alveolar surface tension and hamper alveolar fluid resorption (Table 1) [67,68,69,70,71]. Thus, treatments to alveolar edema may help to both reduce the size of poorly ventilated area and increase the blood oxygen content (Figure 1). Although exogenous surfactant replacements in animal models of ARDS and neonatal respiratory distress syndrome (RDS) showed consistent improvement in gas exchange and survivals, some adult studies have shown only improved oxygenation but no survival benefits [70]. Moreover, a few exogenous pulmonary surfactants have been currently authorized to treat pulmonary permeability edema in COVID-19 patients [70].

Besides exogenous surfactant replacements, other treatments that counter-balance the inhibition of edema clearance during hypoxia or improve the lung’s ability to clear alveolar edema should also be adopted. For example, a relatively high partial pressure of O_2_ in the alveolar gas facilitates alveolar fluid resorption by activating Na^+^ transport across the alveolar epithelium, which makes an osmotic gradient responsible for the lung edema clearance [66]. Hereby, appropriate oxygen inhalation would accelerate the resorption of pulmonary edema fluid. However, simple oxygen therapy would not achieve adequate effects on alveolar edema clearance. Ethanol/butanol-oxygen vapor therapy (oxygen inhalation with 20% ethanol or butanol as humidifying agent) is a common treatment to pulmonary edema with a long history of safety [72,73,74,75]. Ethanol/butanol vapor decreases the surface tension of the foam inside the pulmonary alveoli and therefore alleviating alveolar edema (Table 2) [72,73,74,75]. Moreover, conservative fluid management, in which diuretics may be administered and intravenous fluid administration is minimized, would reduce hydrostatic pressure and enhance serum oncotic pressure, and therefore limit the development of pulmonary edema potentially (Table 2) [76,77,78]. Unfortunately, these common therapies to pulmonary edema have not attracted enough attention in COVID-19 clinical practice.

Autopsy showed that pulmonary fibrosis was not serious in dead patients with SARS-CoV-2 infections. Intact alveoli could still be seen, but exudation was serious [64,65,79,80,81,82], suggesting the severe alveolar edema. COVID-19 has a prominent feature, that is, a large amount of mucus (edema fluid) could be found in the small airway [64,65,79,80,81,82], which is distinct from other acute pulmonary injuries.

When the disease develops into late stages, systemic alveolar edema and severe ventilation-perfusion mismatch occur, and the blood oxygen will decline sharply. Then the high flow oxygen therapy and mechanical ventilation will be required (Figure 1). However, high-flow oxygen inhalation inhibits HPV because of the reversed diffusion of oxygen, that is, if enough oxygen could bind the receptor in the small alveolar-capillary-arteriole space, the vessels will not vasoconstrict [83]. Upon a high-flow and high-concentration oxygen inhalation, the reflex stimulation to respiration by hypoxia will disappear, resulting in a more serious retention of CO_2_, which may lead to the hypercapnic encephalopathy (hypercapnic coma) or even a respiratory arrest [35]. Furthermore, high inspired oxygen concentrations could lead to pulmonary-specific toxic effects, such as denitrogenation phenomena, inhibition of surfactant production and severe acidosis, and therefore may worsen ventilation-perfusion mismatch or induce a degree of hypoventilation (Table 2) [84,85]. On the other hand, mechanical ventilation may cause mucus to be blown into the deep of the small airways, which then aggravates intrapulmonary shunt and alveolar hypoxia (Table 2) [86]. These deleterious effects may be an important reason for the high mortality after high-flow oxygen inhalation and mechanical ventilation (The ICU mortality rate among those who required non-invasive ventilation was 79% and among those who required invasive mechanical ventilation was 86%.) [9]. Therefore, nebulized heparin treatments, ethanol-oxygen vapor therapy should be given at the early stages of COVID-19.

## 5. The Optimal Time and SpO_2_ Threshold for Oxygen Therapy

In a retrospective case report that included 69 adults in Wuhan, China, 29% of patients showed dyspnea and 20% of cases (14 patients) showed oxygen saturation SpO_2_ <90% [oxygen index (OI) < 110 mmHg] during admission [87]. In their report, as of 4 February 2020, 18 (26.9%) of 67 patients had been discharged, and five patients had died, with a mortality rate of 7.5%. Noticeably, all five deaths occurred in the SpO_2_ <90% group [87]. The median time from onset of symptoms to admission was six days (inter quartile range 4–9 days). However in the SpO_2_ <90% group, the median occurrence time of lowest SpO_2_ was only one day (inter quartile range 0–2 days) after admission [87]. In other words, SpO_2_ of some patients at admission were already very low, which may develop severe ARDS subsequently (Table 3). Therefore, it may be too late for the patients to take oxygen therapy after admission. The best window period of oxygen therapy may be the six days from onset of symptoms to admission.

Dai et al. [88] classified COVID-19 patients into four stages according to the CT (Computed tomography, which uses X-rays to produce cross-sectional images) performances. Stage I: one or more lesions, in irregularly patchy or round shapes, generally showing ground-glass opacity with vascular enlargements. Stage II: more area lesions, found in bilateral lobes mainly at the sub-pleural areas, in irregularly patchy, round or reverse-butterfly shapes, diffused or scattered patches occasionally fusing into a large patch with a high density, vascular enlargements, reticular signs and bronchial wall thickening, sometimes with little fibrosis and atelectasis in sub-segments. Stage III: some lesions diminished or absorbed, the focus could be entirely absorbed, showing residual fibers. Stage IV: bilateral diffuse inhibitions, over half of the lung areas involved, occasionally extended to the entire lung and defined as the white lung, implying the systemic alveolar edema. However, the patients in the stage IV group showed only slightly-declined SpO_2_ (94.70 ± 0.20%). While the stage II patients with only a small proportion of lung injury and HPV showed relatively high SpO_2_ (97.2 ± 0.91%) [88]. SpO_2_ <95% may indicate late infection stages (Table 3) [88].

Moreover, a recent study demonstrated a high correlation between decreased SpO_2_ and severe cases that 78.0% (32/41) of the patients with SpO_2_ ≤95% would develop into severe diseases (Table 3) [89]. The risk threshold of SpO_2_ was 95%.

WHO and BMJ Best Practice suggested SpO_2_ ≤90% or signs of severe respiratory distress, central cyanosis, shock, coma and/or convulsions as diagnostic standards for the severe pulmonary infections [90,91]. In addition, according to the novel coronavirus pneumonia diagnosis and treatment plan (trial version 7) published by the National Health Committee of China, either respiratory rate (RR) ≥30 times per minute, or resting state SpO_2_ ≤93%, or OI ≤300 mmHg was defined as the severe condition, and then the oxygen therapy was given (Table 3) [92]. However, the median SpO_2_/respiratory rate value was significantly higher in COVID-19 patients than in non-COVID-19 patients, which implies that a normal breathing rate could mask profound hypoxia and make severity assessment in COVID-19 patients more difficult in out-of-hospital settings [93]. Besides, based on the above analysis, the diagnostic standard of SpO_2_ either ≤90% or ≤93% may be too low to take the oxygen therapy in time.

## 6. Four Clinical Comments

Based on the above analysis, we propose four comments to prevent SARS-CoV-2 patients from developing into severe hypoxemia:

(a) For the suspected cases with symptoms, finger SpO_2_ (with finger oximeter ideally) should be measured at each time of nucleic acid test sampling and daily after symptom onset. However, finger SpO_2_ varies greatly with the altitude and the age [35], and the finger oximeter itself may have a large deviation, so it is recommended that each oximeter should be calibrated with several healthy people of different ages to get the reference value. If the patient’s SpO_2_ was lower than the reference value by 3% or more (e.g., if the reference value was 98%, then ≤95% is the threshold for oxygen therapy), it is suggested that the patients were hospitalized immediately for standard low-flow oxygen inhalation possibly combined with 20% ethanol as humidifier. If immediate hospitalization was not possible, the patient was recommended to take oxygen in the home, such as with a portable oxygen respirator. During the in-home oxygen therapy, finger SpO_2_ should be monitored continually to assure that SpO_2_ has been restored to 96%, but not higher than that (Table 3). This is because saturation above this level likely causes an increased risk of death without plausible benefits [94,95]. This upper limit may be lower for the patients with chronic respiratory diseases. For instance, the oxygen treatment goal should be 88–92% for patients with chronic type II respiratory failure (Table 3) [92]. Nevertheless, if SpO_2_ cannot be enhanced afterwards, the patient should seek medical advice or go to the hospital in time. The in-home oxygen therapy may be of great significance for countries with a shortage of medical resources. The COVID-19 patients usually require oxygen long-term oxygen supplies. However, if humidification with 20% ethanol was adopted, long-time ethanol vapor inhalation may generate adverse effects to the respiratory system and the nervous system [96,97]. The optimal length of ethanol-oxygen vapor therapy needs to be investigated in clinical trials.
(b) For the patients with very low SpO_2_, high-flow oxygen inhalation should be applied. Nevertheless, humidification with 20% ethanol might be also recommended on this occasion.
(c) Fluid management might be considered for all COVID-19 patients and conservative fluid management might be applied to severe cases. Some patients may be dehydrated with evolving acute kidney injury at hospital presentation for COVID-19 pneumonia. Therefore, conservative fluid management to these patients should be applied with caution. Detailed guidance of fluid administration in patients with COVID-19 has been discussed elsewhere [98]. For the in-home patients, appropriate reduction in water intake might be an expedient measure.
(d) The prone position could reduce the risk of ventilation-associated lung injuries by the combined effects of more uniform distribution of breathing and less compression of the left lower pulmonary lobe by the heart [5,99,100,101]. Therefore, patients with low SpO_2_ are advised to use prone position as much as possible. In addition, the patients should avoid any vigorous activity that may increase respiratory rate and tidal volume because pulmonary injury will be worsened by the mechanical stretch during the strained breathing [5,99,100,101]. The benefit of prone ventilation is larger than that for typical ARDS. HPV is regionally variable, resulting in heterogenous ventilation-perfusion matching. Prone ventilation may minimize the heterogeneity and allow HPV to divert blood flow to the caudal/dorsal regions of the lung. Although HPV is considered to be weak in COVID-19, residual HPV might be optimized when prone [38].

## 7. Clinical Outlook

In summary, we suggest that in the beginning of the COVID-19 pulmonary involvement (decrease of SpO_2_ of 3%) the patients should receive immediate oxygen therapy and pulmonary edema treatments. However, this is merely a concept paper that needs to be tested in controlled randomized trials. It should be noted that not only SpO_2_ is important but also the patient’s associated tachypnea or hyperpnea, and SpO_2_ should be interpreted with caution as there is a left-sided shifting of the oxyhemoglobin dissociation curve due to tachypnea/hyperpnea induced by hypoxemia [102]. Thus, SpO_2_ monitoring with a finger oximeter is just a stop-gap measure, and the CT performance is still a “golden rule”.

Besides ventilation-perfusion mismatch, intravascular coagulation and alveolar edema, COVID-19 ARDS is a very complex disease, with intrapulmonary shunting, impaired lung diffusion, inflammation, etc. [103,104,105,106,107,108,109]. We cannot expect that early oxygen therapy and pulmonary edema treatments can prevent every COVID-19 patient from the development of ARDS. Antiviral drugs, anti-inflammatory agents and anticoagulant therapies (e.g., heparin as mentioned above) should be adopted along with pulmonary edema treatments.

## Figures and Tables

**Table 1:** Pulmonary pathological changes in COVID-19.

Injuries	Pathogenic Mechanisms	Refs.
Alveolar endothelial injury	Endothelial barrier disruption induces intrapulmonary shunt, hypoxia, intravascular coagulation and the release of pro-inflammatory factors.	[1,5,35]
ACE2-decline-induced pulmonary injury	ACE2 deficiency leads to enhanced and protracted tissues, and vessel exposure to Ang II, which then enhances thrombosis and cell proliferation, increases tissue permeability, cytokine production and inflammation.	[25,26,27,28,29,30,31,32,33,34]
Loss of hypoxic pulmonary vasoconstriction (HPV)	HPV directs blood perfusion from badly-ventilated to well-ventilated alveoli to optimize gas exchange.	[36,37,38]
General pulmonary vasoconstriction	Lead to pulmonary hypertension and a risk of right-heart failure subsequently.	[39,40,41]
Severe ventilation-perfusion mismatch	Induce hypoxemia in the non-injured fraction or/and cause hyper-perfusion of the small injured fraction.	[42,43]
Intravascular coagulation and microthrombi formation	Lead to increased wasted ventilation and less efficient carbon dioxide removal.	[44,45,46,47,48,49,50,51,52,53,54,55,56,57,58,59,60,61,62]
Diffuse alveolar damage (DAD)	Lead to hypoxia at the edematous alveoli.	[63,64,65]
Alveolar edema	Lead to great decline in oxygen diffusion over the blood-air barrier (hypoxia); Hypoxia in turn inhibits oedema fluid clearance.	[5,66]
Impaired alveolar surfactant production	Increase alveolar surface tension and hamper alveolar fluid resorption.	[67,68,69,70,71]

**Table 2:** Putative drugs and treatments to SARS-CoV-2 pneumonia.

Drugs or Treatments	Therapeutic Mechanisms	Therapeutic Effects	Refs.
Oxygen inhalation	Oxygenation enhancement	Alleviate hypoxia; however, high-flow oxygen lead to pulmonary toxic effects.	[35]
Mechanical ventilation	Oxygenation enhancement	Alleviate hypoxia; however, mechanical ventilation may cause mucus to be blown deep into the small airways, which then aggravates alveolar hypoxia.	[9]
ECMO	Oxygenation enhancement	Alleviate hypoxia; however, patients receiving ECMO still showed a high mortality rate.	[9]
Inhaled nitric oxide	Decrease pulmonary vascular resistance	Improve ventilation-perfusion ratio; however, may cause further arterial desaturation (hypoxia).	[35,47,48]
Aspirin	Anticoagulation	Reduce ICU cases, but show no apparent association with the fatality.	[49,50,51]
Heparin	Anticoagulation; block virus entry; increase antibody titres	Reduce the risk of in-hospital mortality and decrease the occurrence of severe cases; however, could not completely prevent occurrence of severe cases.	[52,53,54,55,56,57,58,59,60,61,62]
Exogenous pulmonary surfactant	Alleviate alveolar edema	Reduce the mortality of infants with neonatal RDS; however, clinical outcomes for COVID-19 patients need further investigation.	[67,68,69,70,71]
Ethanol–oxygen vapor therapy	Alleviate alveolar edema	May reduce occurrence of severe cases and the mortality rate (need clinical verification).	[72,73,74,75]
Conservative fluid management	Alleviate alveolar edema	May reduce occurrence of severe cases and the mortality rate (need clinical verification).	[76,77]

**Table 3:** Blood oxygen saturation (SpO_2_) lower limit and upper limit for oxygen therapy.

SpO_2_ Limits		Corresponding Disease Stage	Refs.
**SpO_2_ Lower Limit for Oxygen Therapy**	90%	In the SpO_2_ <90% group, the median occurrence time of lowest SpO_2_ was only one day after admission, indicating a very late stage.	[87]
	94.7%	Stage IV: bilateral diffuse inhibitions, over half of the lung areas involved, occasionally extended to the entire lung and defined as the white lung.	[88]
	95%	78.0% of the patients with SpO_2_ ≤95% would develop into severe diseases (late stages).	[89]
	93%	Resting state SpO_2_ ≤93% indicates a severe condition (late stages).	[92]
**SpO_2_ Upper Limit for Oxygen Therapy**	96%	For most patients receiving oxygen therapy.	[94,95]
	88–92%	For patients at risk of hypercapnic respiratory failure.	[94]
	88–92%	For patients with chronic type II respiratory failure.	[92]

## Data Availability

Not applicable.

## Abbreviations

ACE2	angiotensin-converting enzyme 2
AEI	alveolar epithelial cells type I
AEII	alveolar epithelial cells type II
ALI	acute lung injury
Ang II	angiotensin II
ARDS	acute respiratory distress syndrome
COPD	chronic obstructive pulmonary disease
COVID-19	coronavirus disease 2019
CT	computed tomography
DAD	diffuse alveolar damage
ECMO	extracorporeal membrane oxygenation
*F*_i_O_2_	fractional inspired oxygen
GAGs	glycosaminoglycans
HIF-1α	hypoxia inducible factor-1α
HPV	hypoxic pulmonary vasoconstriction
ICU	intensive care unit
IPF	idiopathic pulmonary fibrosis
NO	nitric oxide
*P*_a_O_2_	arterial oxygen tension
PASMC	pulmonary artery smooth muscle cells
PDK	pyruvate dehydrogenase kinase
PH	pulmonary hypertension
PKC	protein kinase C
RAAS	renin-angiotensin-aldosterone system
RBD	receptor-binding domain
ROS	reactive oxygen species
SARS-CoV-2	SARS-like coronavirus 2
Sicam	soluble intercellular adhesion molecule
SpO_2_	blood oxygen saturation
TMPRSS2	trans-membrane protease/serine subfamily 2
VEGF	vascular endothelial growth factor
vWF	von Willebrand factor
